# Ferroptosis regulator FANCD2 is associated with immune infiltration and predicts worse prognosis in lung adenocarcinoma

**DOI:** 10.3389/fgene.2022.922914

**Published:** 2022-10-04

**Authors:** Chenyang Ye, Yier Lu, Zhijun Yuan, Mi Mi, Lina Qi, Ying Yuan, Shanshan Weng

**Affiliations:** ^1^ Department of Medical Oncology, Key Laboratory of Cancer Prevention and Intervention, The Second Affiliated Hospital of Zhejiang University School of Medicine, Hangzhou, China; ^2^ Cancer Center, Zhejiang University, Hangzhou, China; ^3^ Department of Radiation Oncology, Key Laboratory of Cancer Prevention and Intervention, Ministry of Education, The Second Affiliated Hospital of Zhejiang University School of Medicine, Hangzhou, China

**Keywords:** ferroptosis, lung cancer, tumor immune microenvironment, prognosis, FANCD2, theurapeutic target, B7-H3

## Abstract

Lung adenocarcinoma (LUAD) remains one of the leading causes of cancer-related death. Although immunotherapy has been shown to improve survival in LUAD patients, only a select group of LUAD patients could benefit from it. The correlation between ferroptosis and the tumor immune environment requires further investigation in the setting of LUAD. An analysis using The Cancer Genome Atlas (TCGA)-LUAD cohort systematically evaluated the expression levels of ferroptosis regulators between LUAD and normal tissues and demonstrated the correlation of ferroptosis regulators with the immune checkpoint B7-H3 expression. Based on consensus clustering analysis, we divided LUAD patients into two subtypes according to the expression pattern of ferroptosis regulators. Cluster 2 patients showed more favorable overall survival (OS) (*p* < 0.001) and disease-free survival (DFS) (*p* < 0.001) than Cluster 1 patients. CIBERSORT analysis indicated that Cluster 1 patients harbored higher infiltrated levels of uncharacterized cells, CD4^+^ T cells (nonregulatory), and myeloid dendritic cells, while Cluster 2 patients were more correlated with B cells, M1 macrophages, natural killer cells (NK cells) and regulatory T cells (Tregs). More importantly, we identified FANCD2 as a potentially unfavorable prognostic factor that was overexpressed in LUAD and positively associated with the checkpoint molecule B7-H3 expression. In addition, higher FANCD2 expression was related to a higher tumor immune dysfunction and exclusion (TIDE) score, indicating lower responder rates to cancer immunotherapeutics. In summary, our study suggested a relationship between immune infiltration and ferroptosis and that FANCD2 is a potential biomarker for clinical outcomes and a therapeutic target for LUAD therapy concerning ferroptotic regulation. Our findings may help to advance personalized treatment and improve the prognosis of LUAD.

## Introduction

Non-small cell lung cancer (NSCLC) represents 85% of lung cancers and remains one of the leading causes of cancer-related death. Lung adenocarcinoma (LUAD) is the most prevalent subtype of NSCLC and is associated with more than 500,000 deaths each year (1) (2). LUAD is driven by aberrations of Kirsten rat sarcoma virus (KRAS), epidermal growth factor receptor (EGFR), ROS proto-oncogene 1 (ROS1), tumor protein p53 (TP53), serine/threonine kinase 11 (STK11), etc. (3). Despite great improvements in LUAD treatment, with advances in targeted therapies and chemotherapies, only a select group of LUAD patients could benefit from the current therapeutic strategies. Therefore, further research to explore novel targets for LUAD treatment is urgently needed.

In recent years, the emergence of immunotherapy has brought encouraging results in LUAD treatment. Nevertheless, immune checkpoint blockade (ICB) has not benefited all LUAD patients, and the underlying mechanism remains to be investigated (4). It is challenging to elucidate the mechanisms of immune evasion and the crosstalk in the complex tumor immune microenvironment (TIME).

Ferroptosis is one of the forms of nonapoptotic cell death triggered by the excessive accumulation of iron, resulting in lipid peroxidation and oxidative stress (5). The characteristics of ferroptotic cells are mainly morphological changes due to plasma membrane damage (6). Studies have shown that ferroptosis might be an adaptive feature to eradicate carcinogenic or infected cells 7) and that ferroptosis is correlated with many cancer types, such as melanoma (8), pancreatic cancer (9), and lung cancer (10). Emerging evidence suggests that ferroptosis regulators, such as SLC7A11 and NFS1, play vital roles in tumorigenesis and tumor growth in LUAD (11) (10). Impressively, the regulation of ferroptosis is involved in the suppression of tumor progression by enhancing conventional therapeutic efficacy, including chemotherapy and molecular targeted therapy. For example, erastin, as an inducer of ferroptosis, was found to improve the efficacy of cisplatin (12). However, FANCD2, a negative regulator of ferroptosis, was not related to the prognosis of NSCLC patients treated with platinum-based chemotherapy (13). Thus, further investigation about the prognostic and predicative merits of ferroptosis regulators in LUAD is needed. Additionally, the induction of ferroptosis can sensitize epidermal growth factor receptor (EGFR)-activating mutant lung cancer cells resistant to EGFR-tyrosine kinase inhibitors (TKIs) (14). Interestingly, researchers have found that ferroptosis might play a vital role in regulating the TIME. Recently, ferroptotic induction was reported to be the key factor for tumor regression caused by the blockade of programmed cell death-ligand 1 (PD-L1) plus cytotoxic T-lymphocyte associated antigen 4 (CTLA-4) in melanoma (8). Although ferroptosis is pivotal for tumor progression, the role and mechanism of ferroptosis in the TIME of LUAD remain to be elucidated.

Herein, we collected and analyzed the RNA-seq data and corresponding clinical information related to LUAD acquired from The Cancer Genome Atlas (TCGA) and gene expression omnibus (GEO) data portals. First, we classified LUAD patients into two subtypes by consensus clustering analysis and principal component analysis (PCA) of ferroptosis regulators. Then, we explored the correlations of ferroptosis regulators with prognosis, selected immune checkpoints, and infiltrated tumor immune cells. Subsequently, we screened the key ferroptosis regulator shown in the intersection of the Venn diagram and validated the predictive efficacy of FANCD2 for overall survival (OS) by using the “time receiver operating characteristic (ROC)” R package. Finally, Gene Ontology (GO) enrichment and Kyoto Encyclopedia of Genes and Genomes (KEGG) pathway analyses were implemented to study the functional mechanisms underlying ferroptosis regulators in the TIME based on FANCD2 expression. Therefore, this study aimed to explore reliable biomarkers for prognosis or targets for LUAD therapy based on ferroptotic regulation.

## Materials and methods

### Data source and preparation

RNA-seq data and corresponding clinical information related to LUAD were acquired from TCGA data portal (https://tcga-data.nci.nih.gov/tcga/). To further analyze the differential expression of FANCD2, we downloaded the two independent datasets from the GEO database (https://www.ncbi.nlm.nih.gov/geo/): GSE32863 and GSE40275 datasets. The GSE32863 included 116 LUAD individuals and the GSE40275 includes 59 LUAD samples with reliable information.

### The correlation of ferroptosis with prognosis and immune characteristics in subgroups

“ggplot2” and “pheatmap” packages in R software were used for analysis of expression discrepancy between tumor tissues and adjacent pairs in LUAD. To obtain an unambiguous consistency analysis, “Consensus Cluster Plus” (v1.54.0) and PCA were performed to stratify the LUAD patients into two clusters from the TCGA bataset. The survival differences between the two subgroups were evaluated by the software packages “survival” and “survminer” shown as Kaplan-Meier curves with log-rank test to assess OS and DFS. The “CIBERSORT” algorithm was adopted to identify the infiltration of tumor immune cells in two clusters for LUAD patients.

### Screening and identification of the key ferroptosis regulator

To explore the potentially key ferroptosis regulators, Spearman correlation analysis was utilized to demonstrated the association between B7-H3 and 25 selected ferroptosis regulatory genes. Subsequently, Venn diagram was used to identify the key ferroptosis negative regulator FANCD2, which was positively correlated with B7-H3 but negatively linked to survival. The difference analysis of FANCD2 expression level in the adjacent tumor tissues and high/low FANCD2 subgroups was conducted using Wilcox-test and Kruskal-Wallis-test. The R software “forestplot” package was adopted to perform univariate and multivariate cox regression analysis to show the *p* values, hazard (HR) with 95% confidence interval (CI) for each variable. Additionally, predictive efficacy of FANCD2 for 1-year, 3-year and 5-year OS analyses was performed using the “time ROC” R package. Then, the Spearman correlation analysis was employed for elucidating the correlation of FANCD2 with B7-H3, CD274 and mismatch repair (MMR)-related genes, respectively.

### Enrichment analysis

The differential expression of mRNAs analysis was confirmed by the package “Limma” (version: 3.40.2) in R, demonstrated by volcano plots and heat map. The threshold was defined by “Adjusted Log (Fold Change) > 1 or Log (Fold Change) <-1 and *p* < 0.05” (15). We further implemented the GO and KEGG Enrichment Analysis of targeted genes via “ClusterProfier” package.

### Statistical analysis

PCA was performed to stratify the LUAD patients into two clusters from the TCGA dataset. Kaplan-Meier method, Log-rank test were used to evaluate the survival prognosis of patients in LUAD. Wilcoxon test and Kruskal–Wallis test were conducted to analyze the expression difference between groups. Univariate and multivariate Cox analyses were performed to analyze the association between gene expression level and prognosis.

All the above analysis methods and R package were implemented by R foundation for statistical computing (2020) version 4.0.3 and software packages ggplot2 and pheatmap. *p* < 0.05 was considered statistically significant.

## Results

### Expression discrepancy of ferroptosis regulators between LUAD and normal tissues

**FIGURE 1:**
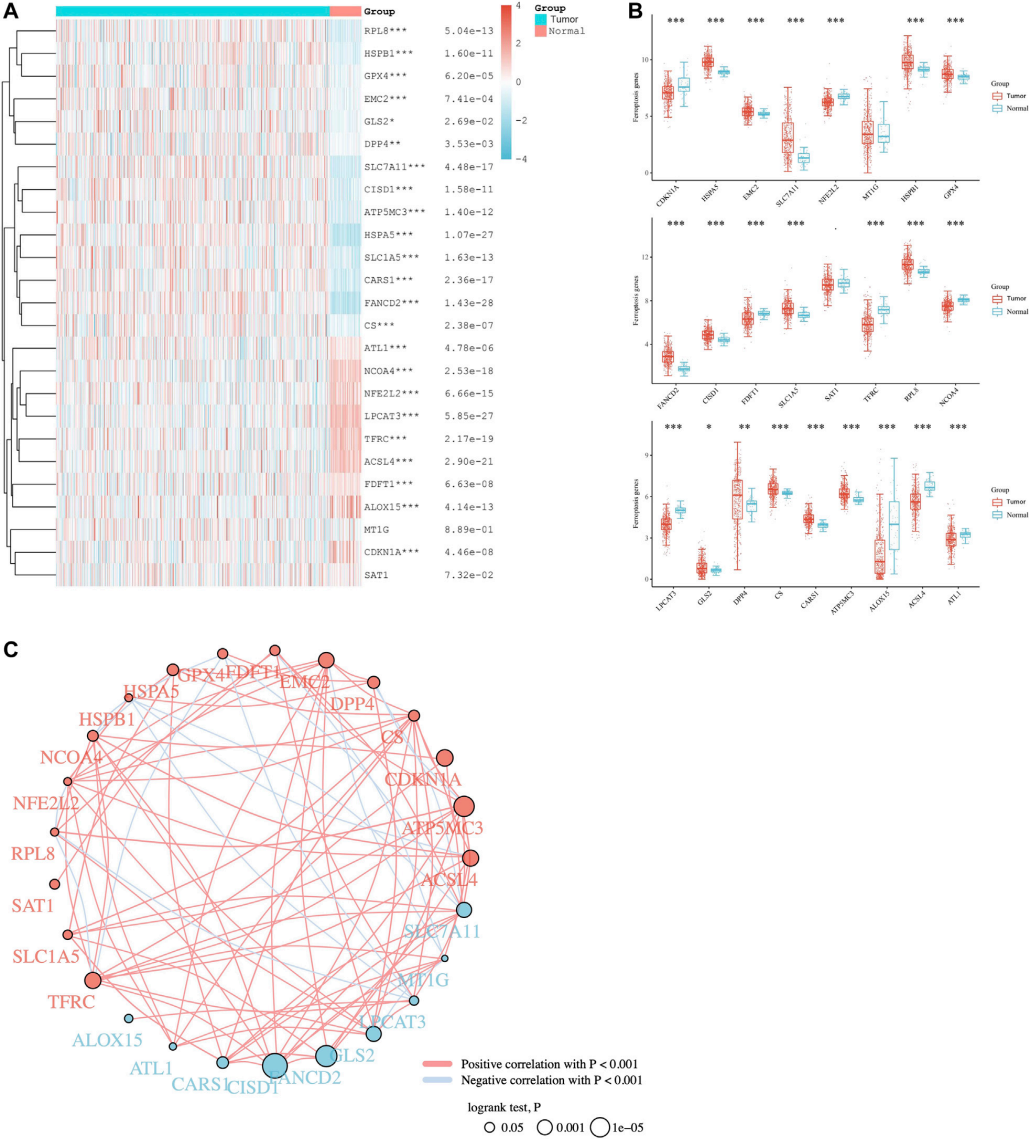
Pan-analysis of ferroptosis regulators in LUAD patients. **(A)** Heat map of ferroptosis regulatory genes between LUAD and normal tissues. **(B)** Wilcoxon Test for analysis of ferroptosis regulatory genes between the two groups. **(C)** The analysis of Spearman correlation between ferroptosis regulators and predictive values of prognosis for LUAD patients. The red and blue dot, respectively represents bad and good prognosis. The red and blue line represents the positive and negative correlation, respectively. The smaller the circle, the larger the prognosis log rank *p*. *p < 0.05, **p < 0.01, and ***p < 0.001.

To explore the underlying functions of the 25 selected ferroptosis regulators in LUAD, the expression levels of ferroptosis regulators in LUAD and adjacent normal tissues were systematically evaluated in the TCGA cohort. The expression profiles in LUAD patients and normal individuals were analyzed, and the two groups showed the differential expression levels of ferroptosis regulators (Figures 1A,B). HSPA5, EMC2, SLC7A11, HSPB1, GPX4, FANCD2, CISD1, SLC1A5, RPL8, DPP4, CS, FANCD21 and ATP5MC3 (all *p* < 0.05) were upregulated in LUAD, whereas the levels of CDKN1A, NFE2L2, FDFT1, SAT1, TFRC, NCOA4, LPCAT3, ALOX15, ACSL4 and ATL1 (all *p* < 0.05) were downregulated. The expression of MT1G and GLS2 (all *p* > 0.05) showed no noticeable divergence. Detailed expression data of these ferroptosis regulators in LUAD patients from the TCGA database are listed in Supplementary Table S1. In addition, based on Liu et al.‘s work, the analysis of Spearman correlation and prognostic values of ferroptosis elucidated that most of these ferroptosis regulators interacted positively and showed crucial prognostic values in LUAD (Figure 1C) (16). The prognostic values were based on survival analysis, which shows the association between ferroptosis-regulated genes (FRGs) and OS in LUAD. These results indicate that ferroptosis regulators might play crucial roles in regulating tumorigenesis and tumor development.

### Prominent differences between two LUAD subtypes regarding the characteristics of ferroptosis regulators and prognosis

**FIGURE 2:**
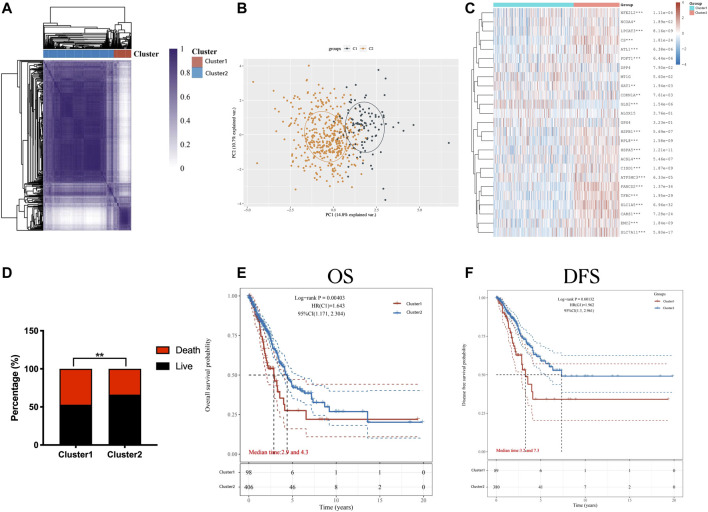
Distinction between the two LUAD subtypes according to the ferroptosis expression and clinical outcomes. Consensus clustering Analysis **(A)** and PCA **(B)** showed the two subtypes divided from LUAD patients. **(C)** The expression levels of ferroptosis regulators in cluster 1/2 subtype in LUAD patients were visualized in the heat map. **(D)** The ratio of live/death in two LUAD subtypes or clusters was shown in the stacked bar chart. **(E,F)** Differences of the OS **(E)** and the DFS **(F)**, as shown in the Kaplan–Meier curves, in the two classified groups. *p < 0.05, **p < 0.01, and ***p < 0.001.

Considering the distinct expression pattern of ferroptosis regulators between the two patient clusters, we first divided the LUAD patients into two subtypes by consensus clustering analysis and PCA, namely, Cluster 1 and Cluster 2 (Figures 2A,B). Unlike Cluster 1 patients, the expression level was upregulated in Cluster 2 patients, involving the majority of ferroptosis regulators (19/25), while the two regulatory genes (GLS2 and SAT1) were downregulated in Cluster 2. In addition, the expression difference of four regulators (DPP4, MT1G, ALOX15, and GPX4) between the two subtypes was not distinct (Figure 2C). We also assessed the prognostic merits of ferroptosis regulators in LUAD. The stacked bar chart (shown in Figure 2D) demonstrates that Cluster 2 patients possessed a higher ratio of live/dead in contrast to the Cluster 1 patients (*p* < 0.05). Detailed clinical information on the two ferroptosis regulator clusters in LUAD is listed in Supplementary Table S2. The Kaplan‒Meier curves of OS and DFS in the two LUAD subgroups exhibited that there was apparent heterogeneity between the two LUAD subtypes or clusters, in which Cluster 2 patients showed more favorable OS (*p* < 0.001) and DFS (*p* < 0.001) than Cluster 1 patients (*p* < 0.05; Figures 2E,F).

### Correlation between ferroptosis regulators and B7-H3 expression level and infiltrated tumor immune cells in LUAD

**FIGURE 3:**
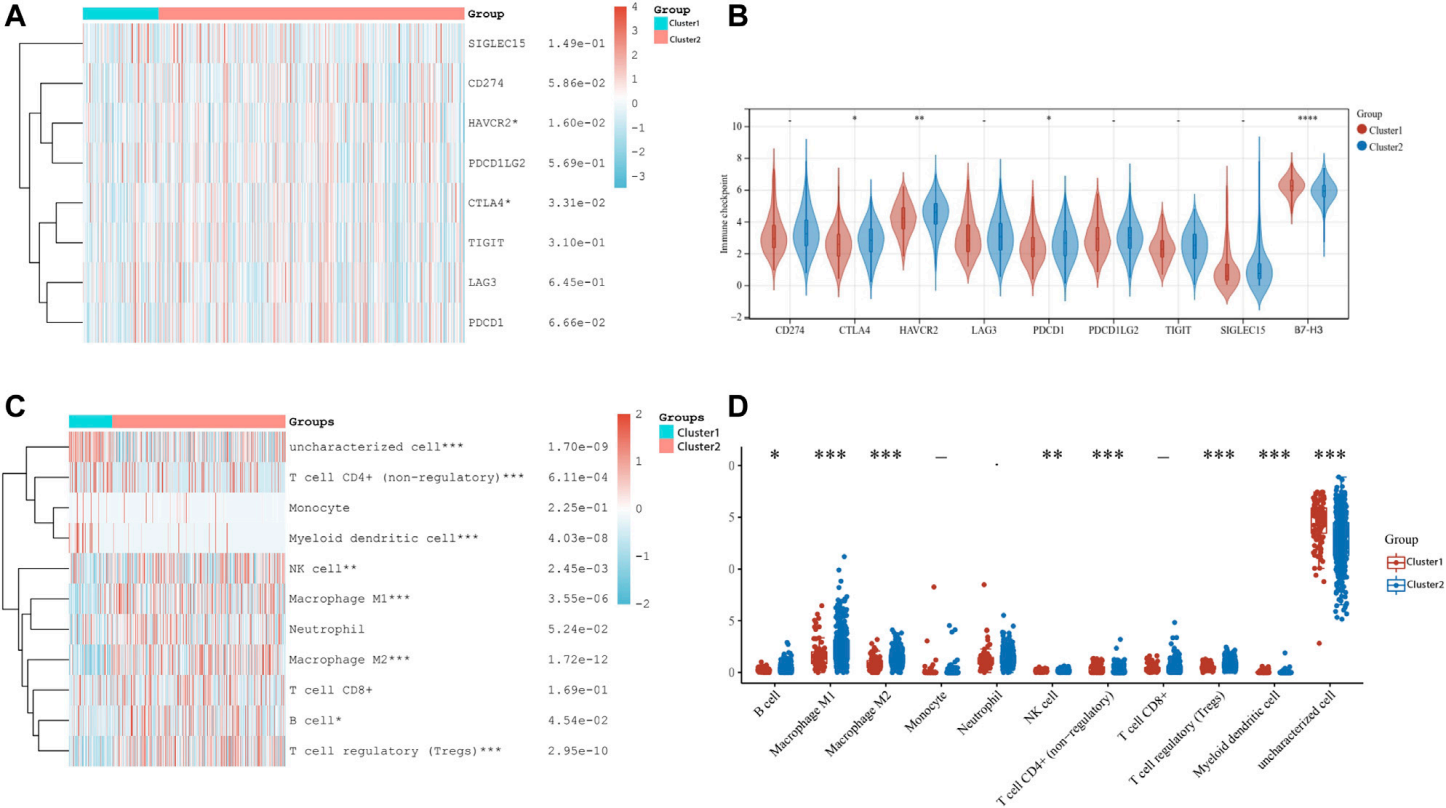
Correlation between B7-H3 expression and ferroptosis regulators and pattern of immune cells infiltration in two subtypes for LUAD patients. **(A)** Heat map of eight common immune checkpoints in cluster 1/2 subtype. **(B)** Wilcoxon Test for analysis of immune checkpoints-related genes in the two clusters in LUAD. **(C)** Heat map visualized the levels of multiple types of infiltrated immune cell in cluster 1/2 in the TCGA-LUAD cohort. **(D)** The results of divergence of infiltrated immune cell types by using Wilcoxon Test for two clusters patients. *p < 0.05, **p < 0.01, ***p < 0.001, and ****p < 0.0001.

Furthermore, a comprehensive correlation analysis between ferroptosis regulators and selected immune checkpoint-related genes (including SIGLEC15, CD274, HAVCR2, PDCD1LG2, CTLA4, TIGIT, LAG3, PDCD1, and B7-H3) was performed (as shown in Figures 3A,B). Detailed expression data of common immune checkpoint molecules in the two clusters are listed in Supplementary Table S3. HAVCR2, PDCD1, and CTLA4 were upregulated in Cluster 2, whereas SIGLEC15, CD274, PDCD1LG2, TIGIT, and LAG3 showed no distinct difference between the two clusters (*p* < 0.05). In contrast to Cluster 1 patients, Cluster 2 patients possessed lower B7-H3 expression. Subsequently, we further identified whether ferroptosis was associated with the TIME in LUAD. The proportion of multiple infiltrated tumor immune cells of LUAD individuals in two clusters was visualized in the heatmap (Figure 3C). Consistently, as shown in Figure 3D, Cluster 1 patients harbored higher levels of infiltrated cells, including uncharacterized cells, CD4^+^ T cells (nonregulatory), and myeloid dendritic cells, while the other 5 cell types were abundant in Cluster 2 patients (M2 macrophages, NK cells, M1 macrophages, B cells and Tregs).

### Crucial ferroptosis regulator FANCD2 is overexpressed in LUAD

**FIGURE 4:**
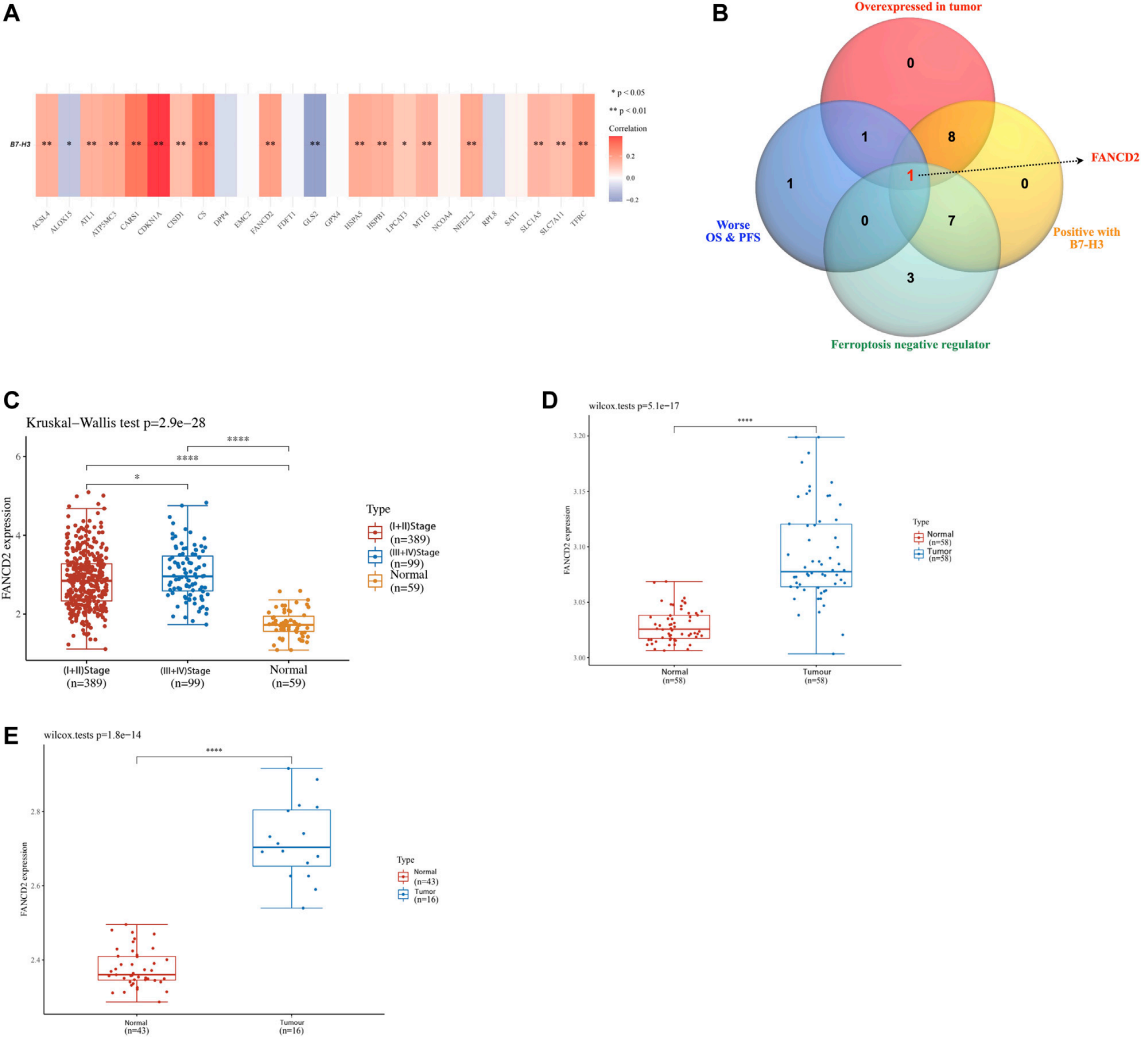
Analysis of the FANCD2 gene expression in LUAD. **(A)** The association of B7-H3 with ferroptosis regulatory genes in LUAD based upon TCGA dataset. **(B)** The Venn diagram demonstrated that the highly expressed FANCD2 was a poor prognostic biomarker and positively linked to the B7-H3 expression in LUAD. The analysis in the TCGA cohort **(C)** and validation sets in GSE32863 **(D)** and GSE40275 **(E)** performed the group comparisons of the expression of FANCD2 in LUAD and adjacent normal tissues, and the correlation of FANCD2 with American Joint Committee on Cancer (AJCC) stages about LUAD. *p < 0.05, **p < 0.01, ***p < 0.001, and ****p < 0.0001.

To investigate the correlation between ferroptosis regulators and B7-H3, an analysis using the TCGA-LUAD cohort was conducted, and the results indicated that most ferroptosis regulators (such as CDKN1A, FANCD21, CS, FANCD2, etc.) were positively linked to B7-H3; however, there was a negative association between ALOX15, GLS2, and B7-H3 (Figure 4A). A detailed correlation analysis of common immune checkpoint molecules and CD276 is listed in Supplementary Table S4. An intersection of ferroptosis regulatory genes upregulated in LUAD, correlated with dismal outcomes, and positively linked to the expression of B7-H3 was used to define key regulators associated with unfavorable clinical outcomes and B7-H3 expression among the 25 selected ferroptosis regulators. The results shown in Figure 4B suggest that FANCD2 was a potentially unfavorable prognostic factor that is overexpressed in LUAD and positively linked to B7-H3 expression. To further investigate the correlation of FANCD2 with LUAD, we analyzed the expression of FANCD2 based on TCGA and GEO datasets. The results indicate that FANCD2 was prominently more overexpressed in LUAD than in adjacent normal pairs in the TCGA cohort and GSE32863 and GSE40275 cohorts (*p* < 0.05, Figures 4C–E). Furthermore, compared with early-stage (stage I-II) patients, the expression of FANCD2 in advanced-stage (stage III-IV) patients was distinctly upregulated (*p* < 0.05, Figure 4C).

### FANCD2 expression with potentially predictive capacity for prognosis

**FIGURE 5:**
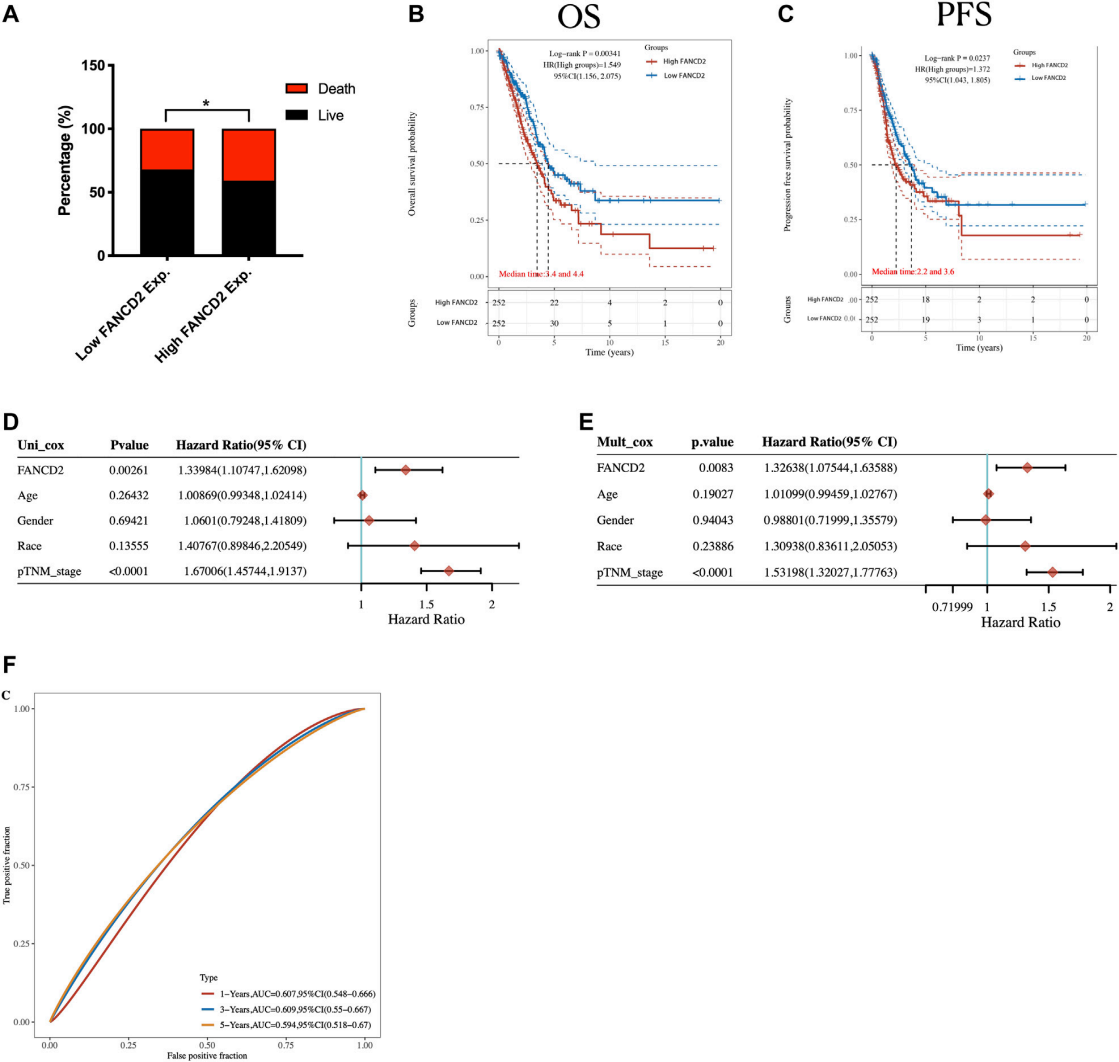
highly expressed FANCD2 is correlated with poor prognosis of LUAD. **(A)** Visualization of the proportion of live/death in high/low FANCD2 groups in LUAD patients. **(B,C)** The Kaplan-Meier analysis based upon TCGA dataset utilized to perform the OS **(B)** and the progression-free survival **(C)** for LUAD patients with high/low FANCD2 expression level. **(D,E)** FANCD2 expression and other factors for prognosis in LUAD patients evaluated by univariate **(D)** and multivariate **(E)** analysis. **(F)** Time-dependent receiver operating characteristics analysis of FANCD2.

We conducted further analyses to evaluate the prognostic value of FANCD2. The stacked bar chart (shown in Figure 5A) illustrates that LUAD patients with higher FANCD2 expression possessed a lower ratio of live/dead than LUAD patients with lower FANCD2 expression (*p* < 0.05). Detailed clinical information and expression data of FANCD2 in LUAD are listed in Supplementary Table S5. Consistent with the results shown in Figure 5A, a Kaplan-Meier analysis demonstrated that compared to LUAD patients with lower FANCD2 expression, those with upregulated FANCD2 expression had unfavorable OS and PFS (Figures 5B,C; Supplementary Figures S1A,B). In addition, a further correlation analysis using the Cox regression model between FANCD2 expression and OS for LUAD patients was carried out. In both univariate and multivariate analyses, FANCD2 expression was defined as an independent biomarker for poor prognosis in LUAD patients (Figures 5D,E). Consistently, the independent prognostic ability of FANCD2 was also confirmed using time-ROC analysis, which showed that the area under the curve (AUC) values of FANCD2 expression for predicting 1-, 3-, and 5-years survival in the ROC curve were 0.607, 0.609, and 0.594, respectively (Figure 5F).

### Analysis of the correlation between FANCD2 expression and B7-H3, immune checkpoint-related genes and tumor-infiltrating immune cells

**FIGURE 6:**
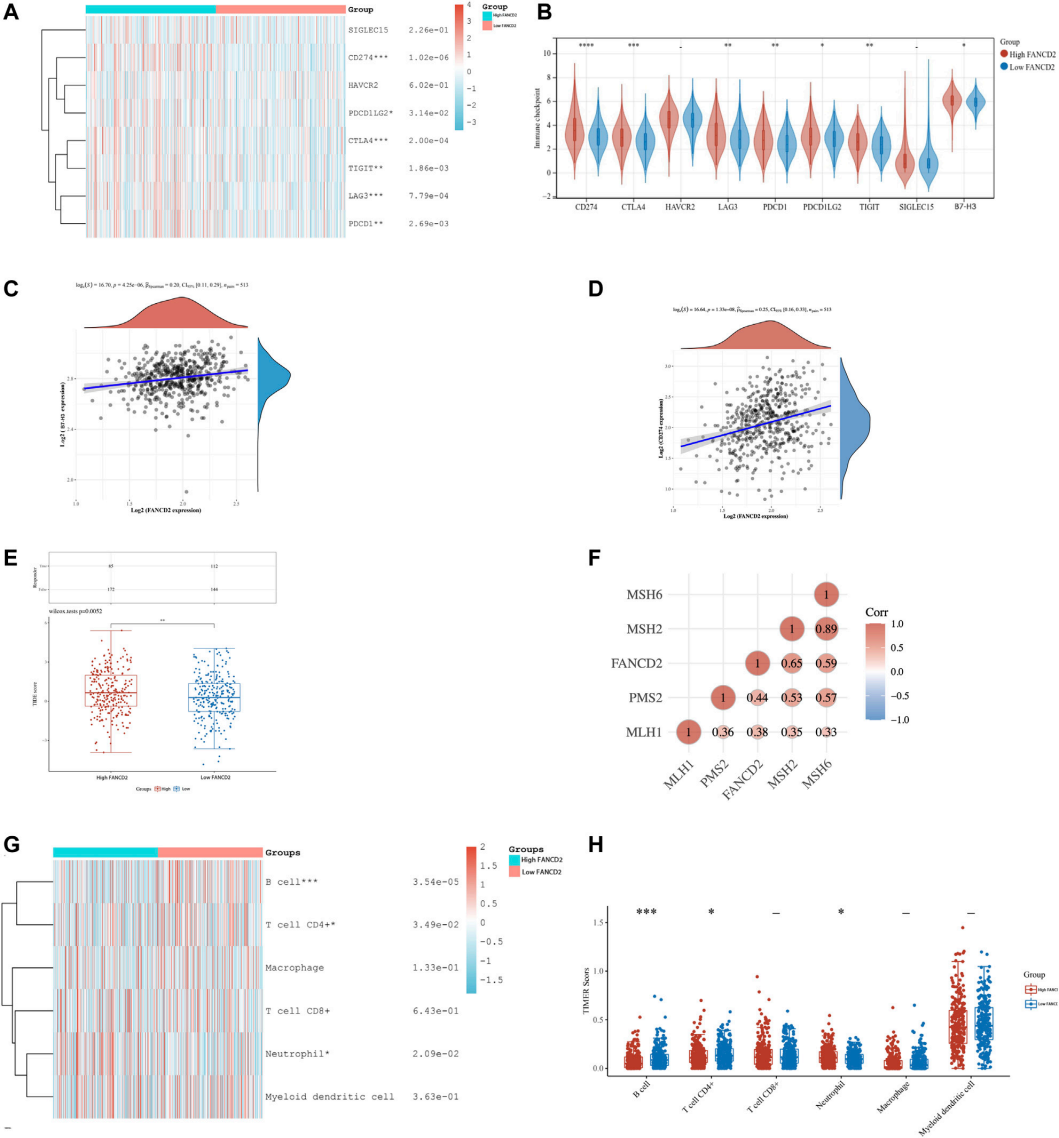
Analysis of correlation between FANCD2 expression and B7-H3, immune checkpoint-related genes and tumor-infiltrating immune cells in LUAD. **(A)** Heat map of eight common immune checkpoints in high and low FANCD2 expression groups in LUAD patients. **(B)** The expression levels of immune checkpoints in the two clusters in LUAD. **(C,D)** Spearman correlation analysis of FANCD2 expression and B7-H3 expression **(C)** and CD274 expression **(D)** in LUAD. **(E)** The analysis of efficacy of immune checkpoint inhibition in the two clusters by calculating the TIDE Score. **(F)** The correlation analysis of FANCD2 and selected four MMR genes. **(G)** Heat map of the infiltrating levels of immune cells in high and low FANCD2 expression groups in LUAD patients. **(H)** The results of differences in abundance of immune cells in the two subtypes. *p < 0.05, **p < 0.01, and ***p < 0.001.

Further expression correlation analysis of FANCD2 with the selected immune checkpoint-related genes was performed (as shown in Figures 6A,B). The results revealed that most of the analyzed genes (such as CD274, PDCD1LG2, CTLA4, TIGIT, LAG3, PDCD1, and B7-H3) were positively correlated with higher expression of FANCD2. Importantly, B7-H3 was overexpressed in LUAD compared with adjacent normal tissues (Supplementary Figure 2A). Additionally, a further correlation analysis showed that FANCD2 expression was significantly positively correlated with the expression of B7-H3 (*p* < 0.001, Spearman = 0.20) and PD-L1 in LUAD (*p* < 0.001, Spearman = 0.25) (Figures 6C,D; Supplementary Figure 2B). To investigate whether FANCD2 is a predictive biomarker for immunotherapy efficacy, we evaluated the relationship between FANCD2 and outcomes by the tumor immune dysfunction and exclusion (TIDE) score (Figure 6E). The results demonstrated that the higher expression level of FANCD2 correlated with a higher TIDE score, indicating lower response rates to cancer immunotherapy. Detailed clinical information and TIDE scores are listed in Supplementary Table S6. Deficient mismatch repair (dMMR) caused by the loss of function of the four main MMR genes could be used as an indicator for a potential response to cancer immunotherapy. Therefore, we analyzed the expression correlation of FANCD2 and four MMR genes and identified that the expression of FANCD2 and these four MMR genes had a significant positive correlation, indicating that higher expression of FANCD2 correlated with lower response rates of cancer immunotherapy LUAD (Figure 6F). Additionally, the heatmap (Figure 6G) visualized the discrepancy of various immune cell subtypes in the TIME of the high and low FANCD2 LUAD groups. As shown in Figure 6H, neutrophils increased in the high FANCD2 expression group, whereas CD4^+^ T cells and B cells decreased (*p* < 0.05).

### In-depth analysis of the key ferroptosis regulator FANCD2 in LUAD

**FIGURE 7:**
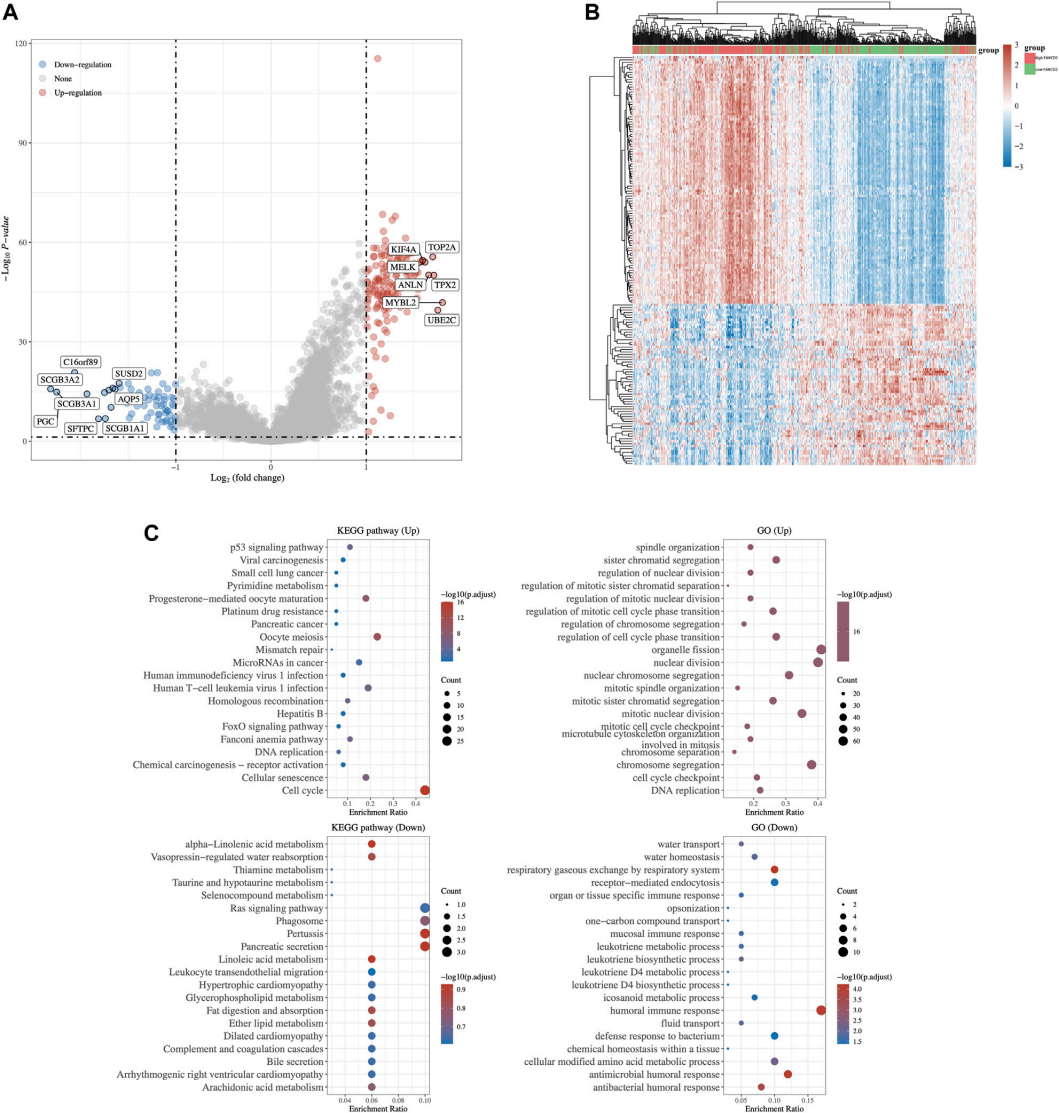
In-depth analysis of key ferroptosis regulator FANCD2 in LUAD. The volcano plot **(A)** and heat map **(B)** visualized the discrepancy of other related genes expression between the high and low FANCD2 expression group in LUAD. **(C)** Analysis of GO term and KEGG pathway using Cluster Profiler package in LUAD individuals with up-regulated expression of FANCD2.

Analyses, including the volcano plot (Figure 7A) and heatmap (Figure 7B), were performed to investigate the genes and potential biological processes that could play crucial roles in LUAD based on FANCD2 expression levels. The results indicate that seven genes were distinctly upregulated with the correlation of high FANCD2 expression: TOP2A, KIF4A, MELK, ANLN, TPX2, MYBL2, and UBE2C, whereas eight genes were downregulated, including SCGB3A2, SCGB3A1, and PGC. Moreover, to further determine the underlying regulatory mechanisms of FANCD2 in LUAD, the Cluster Profiler package was adopted to explore the GO terms and KEGG pathways in the upregulated FANCD2 expression group, as shown in Figure 7C. GO analysis revealed four categories that were positively correlated with highly expressed FANCD2, namely, nuclear division, chromosome segregation, organelle fission, and mitotic nuclear division, while two categories were negatively correlated with highly expressed FANCD2, namely, humoral immune response and antimicrobial humoral response. Additionally, the only pathway positively correlated with FANCD2 expression was the cell cycle, as shown in the results of the KEGG pathway analysis (Figure 7C), whereas three pathways were negatively associated with the expression of FANCD2: phagosome, pertussis, and pancreatic secretion. These findings show that multiple signaling pathways could be involved in the mechanisms causing the TIME discrepancy between the high and low FANCD2 expression clusters, implying that FANCD2 could play pivotal roles in LUAD progression.

## Discussion

**FIGURE 8:**
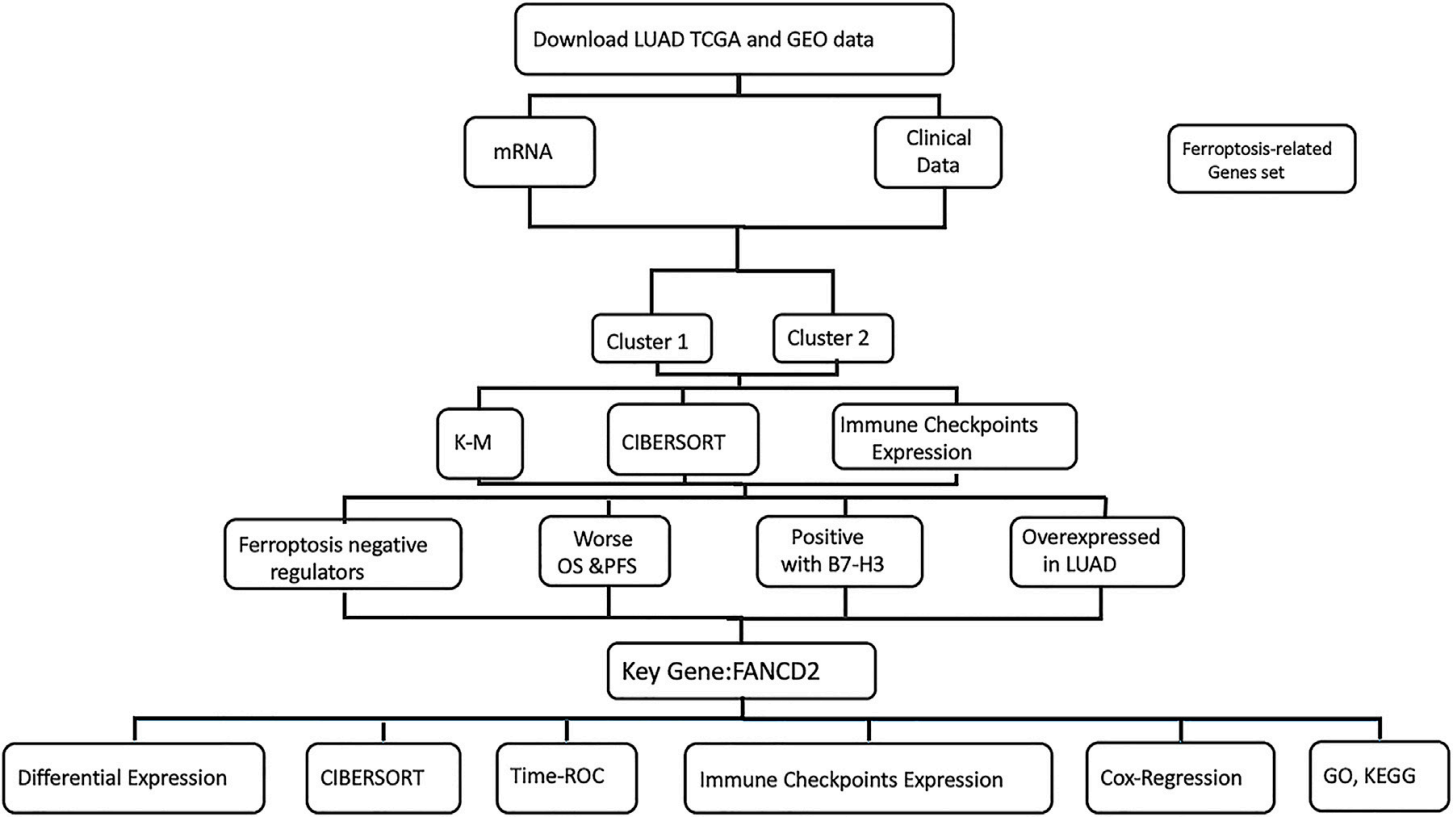
The flowchart to present the logicality of our study.

Herein, we distinguished two independent subtypes with differential clinical features, prognoses, immune checkpoint B7-H3 expression, and TIME of LUAD via consensus clustering for selected ferroptosis regulators (Figure 8). Considering the distinct expression patterns of ferroptosis regulators between the two patient clusters, we first divided the LUAD patients into two subtypes, namely, Cluster 1 and Cluster 2. Among these, FANCD2 was found to be a potentially unfavorable prognostic factor that is overexpressed in LUAD and positively related to immune checkpoint B7-H3 overexpression. Additionally, FANCD2 expression could serve as an indicator of response rates to cancer immunotherapy. Therefore, this study aimed to explore a potential biomarker for prognosis and a therapeutic target for LUAD therapy concerning ferroptotic regulation.

Starting with the TCGA program, precision oncology adapted to individual patients plays a vital role in cancer treatment, which stratifies patients into subtypes by clinically relevant biomarkers (17). For instance, Scheffler et al. classified KRAS-mutated LUAD patients into subgroups according to cooccurring aberrations; and consequently, tumor stage was significantly correlated with the types of additional mutations (18). Based on immunotherapy-associated biomarkers, Li et al. constructed a nomogram for predicting the clinical outcomes of LUAD patients with biological heterogeneity (19). Nonetheless, the specific roles of ferroptosis regulators in LUAD classification remain to be explored. Therefore, we divided LUAD patients into two subtypes by consensus clustering analysis according to the expression pattern of ferroptosis regulators and studied the potential roles of their application in the prognosis and treatment of individuals. We found that Cluster 2 patients possessed a higher ratio of live/dead cells than Cluster 1 patients and that the expression level was upregulated by the majority of ferroptosis regulators.

Further analysis revealed that Cluster 1 patients harbored significantly higher TIDE scores and higher B7-H3 expression than Cluster 2 patients, which implied that Cluster 1 patients possessed unfavorable outcomes of immune checkpoint treatment. B7-H3 is a newly identified checkpoint molecule that plays a crucial role in tumor antigen-specific immune responses and cancer development (20). Upregulation of B7-H3 in multiple cancer tissues, such as cervical and kidney cancers, is generally correlated with dismal outcomes (21). Recent discoveries have shown that B7-H3 is critical in promoting the proliferation, migration, invasion, epithelial-to-mesenchymal transition (EMT), and stemness of tumor cells (22). Furthermore, a phase I trial (NCT02381314, results pending) is ongoing to test enoblituzumab (an anti-B7-H3 mAb) with ipilimumab in NSCLC. The inhibition of B7-H3 signaling to be used alone or in combination with other treatments is expected to contribute to improvements in clinical practice and benefit cancer patients, although validation of the safety and efficacy of targeted B7-H3 treatment remains unconfirmed.

FANCD2 is an essential gene involved in DNA damage repair and ferroptosis inhibition. FANCD2 has been found to induce transcription-dependent and -independent mechanisms, causing iron accumulation and lipid peroxidation and thereby inhibiting ferroptosis in bone marrow injury (23). Fagerholm et al. found that FANCD2 is a sensitive biomarker of favorable prognosis in breast cancer (24). Thus, we aimed to explore the function of FANCD2 as an effective marker for prognosis and therapy. Meanwhile, the data showed that the level of FANCD2 expression is negatively correlated with OS and PFS in LUAD patients. In addition, we analyzed the correlation of FANCD2 with four MMR-related genes (MSH2, MSH6, PMS2, and MLH1) in LUAD, and previous studies of the relationship between ferroptosis regulators and microsatellite instability-high (MSI-H) remain scarce. Therefore, the detailed mechanisms of the crosstalk between FANCD2 and TIME remain to be explored. While studies investigating the relationship between ferroptosis regulators and the expression of CD274 and B7-H3 are limited, our analysis demonstrated that FANCD2 is closely related to the expression of immune checkpoint genes (especially the expression of B7-H3 and CD274). This finding also implies that FANCD2 is a potential target for cancer immunotherapy and might further facilitate the understanding of the mechanism of immunotherapy.

In recent years, several studies have revealed the interaction between ferroptosis and the tumor microenvironment. Yee et al. suggested that neutrophils can induce tumor cell necrosis in glioblastoma progression by promoting tumor ferroptosis (25). In addition, ferroptosis is involved in regulating CD4^+^ T-cell activation in gastric cancer (GC), correlated with prognosis, and these findings provide a novel idea for GC immunotherapy and hold promise for future clinical application (26). Herein, we demonstrated the overexpression and unfavorable prognostic value of FANCD2 in multiple ways. Furthermore, CIBERSORT analysis revealed that the levels of neutrophils were increased in the high FANCD2 expression group, whereas the levels of CD4^+^ T cells and B cells were reduced. This implied a possible regulatory role of FANCD2 in the polarization of tumor-associated macrophages (TAMs). Notably, significantly high infiltration levels of B cells were observed in the low FANCD2 expression group. Therefore, FANCD2 might play a pivotal role in the regulation of the TIME in LUAD.

However, the analysis of the correlation of ferroptosis regulators with TIME was conducted only based on data from TCGA and GEO databases. Therefore, further research to validate the relationship is warranted. Some preclinical models, including cancer organoids cocultured with immune cells and mouse models, would be great options for drug-sensitivity tests. Clinical cohort studies could be helpful to evaluate the prognostic merits of ferroptosis regulators. Additionally, body mass index (BMI) data would be interesting to add into logistic regression analyses. While BMI data are not available in TCGA, it is necessary to investigate the relationship between BMI and ferroptosis in future studies. In addition, the problem of tumor heterogeneity remains intractable and may require further analysis at single-cell resolution.

In summary, our study analyzed the correlation of ferroptosis regulators with prognosis, B7-H3, and infiltrating immune cells in LUAD. Two independent subtypes were also established by consensus clustering for ferroptosis regulators. Obvious tumor heterogeneity, distinct B7-H3 expression, and TIME were observed between the two subtypes, which will contribute to the risk stratification and precision therapy for patients with LUAD. Among the selected ferroptosis regulators, FANCD2 was subsequently identified as an independent prognostic indicator of LUAD patients and positively correlated with B7-H3 expression. Further studies on FANCD2 in LUAD will provide a new perspective on the correlation of FANCD2 with TIME and diverse strategies for cancer immunotherapy.

## Data Availability

The datasets presented in this study can be found in online repositories. The names of the repository/repositories and accession number(s) can be found in the article/Supplementary Material.
