# Supplementary figures and images for: Ferroptosis regulator FANCD2 is associated with immune infiltration and predicts worse prognosis in lung adenocarcinoma
Source: Front Genet. 2022 Oct 4;13:922914. doi: 10.3389/fgene.2022.922914 (PMC9576926; doi:10.3389/fgene.2022.922914)

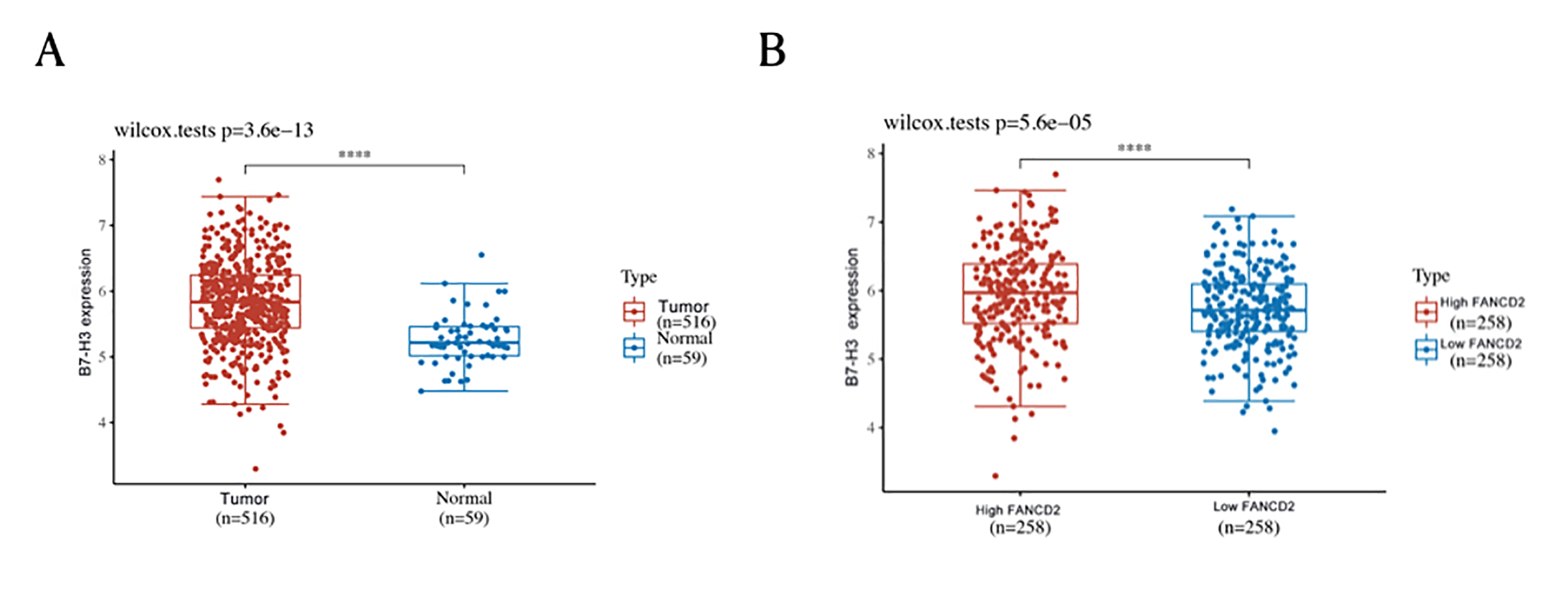

Supplement: Supplementary file 5 [file Image2.TIF]

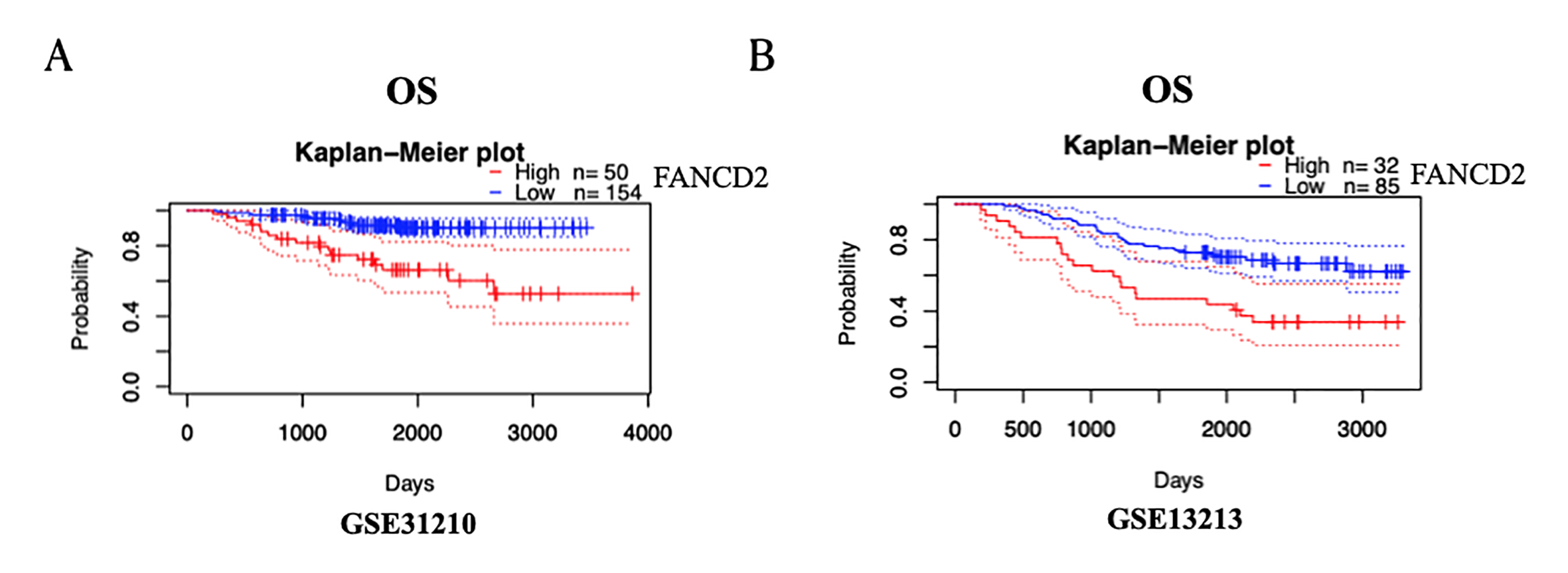

Supplement: Supplementary file 7 [file Image1.TIF]
